# AI and the double competence gap in dental education: towards symbiotic intelligence

**DOI:** 10.3389/fdmed.2026.1965756

**Published:** 2026-10-29

**Authors:** Rune Johan Krumsvik, Kristin Klock, Magnus Bratteberg

**Affiliations:** 1 Department of Education, Faculty of Psychology, University of Bergen, Bergen, Norway; 2 Department of Clinical Dentistry, Faculty of Medicine, University of Bergen, Bergen, Norway

**Keywords:** AI literacy, artificial intelligence, competence gap, dental education, professional digital competence, symbiotic intelligence

## Introduction

Artificial intelligence (AI) is increasingly becoming part of the knowledge infrastructure of healthcare. From diagnostic support and image interpretation to patient communication and clinical decision-making, AI-supported technologies, including generative AI, are changing the conditions under which healthcare professionals work. Dentistry is no exception. Recent research highlights emerging applications of AI in dental practice, while international reviews suggest growing opportunities and challenges associated with integrating AI into dental education. However, the rapid development of AI-supported technologies raises questions about how effectively professional education and continuing professional development can respond to evolving competency requirements. This raises a central question: *How can dental education and continuing professional development respond to the potential double competence gap associated with AI-enabled dental practice?*

In this Opinion article, we address this question by proposing a conceptual framework that distinguishes between an initial education–practice gap and a continuing professional competence gap. Drawing on the concepts of AI literacy, Professional Digital Competence, and symbiotic intelligence, we discuss how dental education may develop and assess the competencies required for critical, ethical, and responsible AI use while preserving independent clinical reasoning, professional accountability, and patient-centred care.

### AI as a moving target: can conventional education models keep pace?

The rapid development of artificial intelligence raises important questions about how dental education can maintain the relevance of its curricula while safeguarding established standards of clinical competence, patient safety, and professional accountability. Although international reviews indicate growing interest in AI applications within dental education, systematic curricular integration appears to vary across institutions and educational contexts (1–3). Such variation should not necessarily be interpreted as resistance to technological change, as cautious implementation may also reflect legitimate concerns about educational quality, clinical validity, ethical safeguards, and the availability of robust evidence. Preliminary analyses of regulations and national curriculum frameworks for professional education raise questions about whether current educational provision adequately addresses emerging AI-related competency requirements, potentially contributing to the two dimensions of the proposed double competence gap.

When generative artificial intelligence became widely accessible through platform-based services such as ChatGPT in the autumn of 2022, many assumed that it would primarily affect the technology sector. However, when we tested a professional version of ChatGPT (GPT-4) in medicine, and partly in dental and psychology education, in March 2023, it was already apparent that this represented a technological paradigm shift with potentially far-reaching implications across professional education programmes (4, 5).

While AI capabilities have developed rapidly in recent years in the health sciences (1, 2, 6–12), their integration into dental education presents a different set of challenges. Curriculum development, faculty preparation, assessment design, and clinical governance require careful consideration and cannot necessarily follow the pace of technological innovation. Recent reviews indicate that AI-related educational initiatives are emerging, but their integration into formal curricula and assessment practices remains variable. Several key explanations emerge from the literature: already overcrowded curricula, insufficient profession-specific digital competence among educators, and a lack of structured implementation (1–3). This potential misalignment between technological developments and structured educational provision provides an important rationale for examining the proposed double competence gap.

Dental education has previously incorporated technologies such as digital radiography, simulation, intraoral scanning, and computer-aided design and manufacturing (13). These developments offer relevant lessons for AI integration: new technologies require faculty development, opportunities for supervised practice, alignment with clinical tasks, and assessment of what students can actually do. However, the current AI transition differs in its breadth and pace. Generative AI can be used across clinical reasoning, communication, learning, and assessment, including through tools that students access independently of their institutions. Moreover, the capabilities and limitations of these systems may change within a single curriculum cycle. Dental programmes should therefore draw on established approaches to curricular implementation while developing a more continuous process for reviewing AI-related learning outcomes, teaching cases, and assessment practices.

### AI in national curriculum frameworks: opportunities and limitations

The Norwegian national curriculum regulations for healthcare professional education (RETHOS) emphasise that educational programmes should respond to society's current and future competence needs. However, AI is not explicitly addressed in the national curriculum regulations for dentistry, medicine, or psychology (14–16). Similarly, the Norwegian Agency for Quality Assurance in Education (NOKUT)'s national evaluation of medical education in 2025 provides limited explicit discussion of AI (17).

These observations should be interpreted with caution. The absence of explicit AI terminology in national regulations or evaluation reports does not necessarily imply that AI-related competencies are absent from institutional curricula, teaching practices, or assessment arrangements. National frameworks may deliberately emphasise enduring, technology-neutral professional competencies, allowing educational institutions to adapt their programmes to technological developments without requiring frequent regulatory revisions.

Nevertheless, the limited explicit attention to AI in these national documents raises relevant questions about how emerging AI-related competencies are interpreted, operationalised, and assessed within individual educational programmes. International reviews have identified variation in the integration of AI into dental education, including challenges related to curriculum development, faculty preparedness, and the assessment of AI-related competencies (1–3).

From the perspective of the proposed double competence gap framework, the central concern is therefore not simply whether AI is explicitly mentioned in national policy documents, but whether educational programmes systematically develop and assess the competencies required for responsible AI use in dental practice. Addressing this question requires empirical examination of institutional curricula, learning outcomes, educational activities, and assessment practices rather than relying solely on national policy analysis.

Although the policy examples discussed here are drawn from Norway, the distinction between national competency frameworks, institutional curricula, and actual educational practices may also be relevant internationally. However, the extent to which the proposed double competence gap applies across different countries and dental education systems requires context-specific empirical investigation.

The increasing integration of AI into healthcare knowledge infrastructures may also have epistemological implications for professional education. As AI systems become involved in information retrieval, diagnostic support, and clinical decision-making, questions arise concerning how professional knowledge is produced, evaluated, interpreted, and applied. These developments may gradually reshape aspects of professional roles and clinical reasoning, although their scope, pace, and educational consequences remain uncertain. Rather than predicting a specific timeline for such changes, dental education should develop adaptable approaches that enable students and practising professionals to critically evaluate emerging technologies while maintaining established standards of clinical expertise and professional accountability (18, 19). Professional digital competence and AI literacy should become integral components of both the knowledge infrastructure and professional competence, and conceptual frameworks need to be developed that account for profession-specific AI use across both urban and rural patient contexts (20).

At the same time, the challenge extends far beyond today's dentist education. The vast majority of today's practising professionals completed their education long before generative artificial intelligence became part of professional practice. Consequently, they have not necessarily received systematic training in how such AI systems can be used professionally, what their limitations are, or which ethical and legal challenges they may entail.

### The double competence gap in dental education

We propose the concept of a *double competence gap* to describe two distinct but interconnected potential mismatches between the competencies required for responsible professional practice in increasingly AI-enabled dental healthcare and the competencies systematically developed through initial dental education and continuing professional development. A competence gap is understood here as a discrepancy between demonstrable professional capabilities and the knowledge, skills, and critical judgement required to perform relevant professional tasks safely, effectively, and ethically. Importantly, such gaps cannot be inferred solely from the availability of new technologies or the absence of explicit AI terminology in educational policy documents; their existence and extent require empirical investigation.

The *first competence gap,* which we term the *initial education–practice gap*, concerns the potential misalignment between undergraduate dental education and the evolving competencies required in AI-enabled dental practice. International reviews suggest that the systematic integration of AI-related learning outcomes, teaching activities, and assessment practices in dental education remains uneven (1–3). This raises questions about whether existing educational provision adequately prepares students for the critical and clinically appropriate use of AI in future dental practice. Although students may independently engage with generative AI and other AI-supported technologies, such engagement does not necessarily constitute systematic development of profession-specific AI literacy. The central educational concern is therefore whether curricula, learning outcomes, teaching practices, and assessment arrangements adequately prepare students to critically evaluate AI-generated information, recognise its limitations, and integrate appropriate AI-supported decision-making with established clinical knowledge and professional judgement.

The *second competence gap*, termed the *continuing professional competence gap*, concerns practising dentists whose initial professional education preceded the widespread availability of contemporary generative AI and other emerging AI applications. These professionals may encounter new technological expectations without having had equivalent opportunities for structured education in AI evaluation, clinical applicability, patient safety, data protection, and professional accountability. However, the extent of this gap is likely to vary across practitioners, clinical settings, previous digital experience, and access to continuing professional development.

The conceptual contribution of the double competence gap framework lies in connecting these two educational challenges across the professional life course. Whereas AI literacy describes a set of relevant competencies, and Professional Digital Competence provides a broader perspective on technology-related professional capabilities, the double competence gap framework draws attention to the potential misalignment between competency requirements and educational provision at two different stages of professional development. It thereby offers a basis for examining how initial education, curriculum and assessment, faculty development, and continuing professional development can respond to changing professional requirements.

We present the double competence gap as a conceptual and empirically testable proposition rather than as an established finding across dental education. Its educational significance depends on whether these proposed mismatches can be identified, assessed, and addressed in specific institutional and professional contexts.

Empirically investigating the proposed double competence gap requires distinguishing between shortcomings in educational provision and demonstrable deficiencies in professional competence. The initial education - practice gap could be examined through curriculum mapping, IADT-guidelines (21), analysis of intended learning outcomes and assessment practices, interviews with educators and students, and performance-based assessments of AI-related clinical competencies. The continuing professional competence gap could be investigated through research informed knowledge (22), surveys, interviews, and scenario-based assessments involving practising dentists with different levels of professional experience and AI exposure. Importantly, the absence of explicit AI-related learning outcomes or structured training would indicate a potential gap in educational provision, but would not, in itself, demonstrate insufficient professional competence. Establishing a competence gap would require comparing observable performance with independently defined, clinically relevant competency standards, while accounting for differences in professional roles, practice contexts, and access to continuing professional development.

## Discussion

### AI literacy, professional digital competence, and symbiotic intelligence

Addressing the proposed double competence gap requires conceptual clarity regarding AI literacy, Professional Digital Competence (PDC), and symbiotic intelligence (SI). Although these concepts are closely related, they represent distinct dimensions of professional competence and human - AI interaction in dental education and practice.

*AI literacy* refers to the knowledge, skills, and critical judgement required to understand, evaluate, and appropriately use AI systems within specific professional contexts. In dentistry, this includes understanding the capabilities and limitations of AI, critically evaluating AI-generated information against clinical findings and relevant evidence, recognising potential errors and algorithmic bias, protecting patient information, and maintaining professional accountability. AI literacy therefore extends beyond technical proficiency to encompass clinical reasoning, ethical awareness, and informed decision-making.

*Professional Digital Competence (PDC***)** represents a broader, context-sensitive dimension of professional competence encompassing the critical, ethical, pedagogical, and clinically appropriate use of digital technologies. Within dentistry, PDC includes established digital practices such as digital radiography, electronic patient records, intraoral scanning, and digitally supported clinical workflows, as well as emerging AI applications. AI literacy can therefore be understood as a specialised and increasingly important component of PDC, rather than as an interchangeable concept.

*Symbiotic intelligence (SI)* provides a complementary conceptual perspective on how human professional expertise and AI capabilities may interact. Building on our previous work on symbiotic intelligence in dental trauma diagnostics (23), SI emphasises the potential for AI to augment, rather than replace, human clinical reasoning and professional judgement. In this perspective, AI may support information processing, pattern recognition, and the exploration of diagnostic alternatives, while dental professionals remain responsible for contextual interpretation, critical verification, ethical considerations, patient communication, and clinical decisions. Importantly, symbiotic intelligence should be understood as a proposed approach to human-AI collaboration, not as evidence that AI-supported decisions necessarily outperform independent professional judgement.

These three concepts have complementary functions within the proposed double competence gap framework. PDC provides the broader professional competency context, AI literacy specifies the AI-related capabilities required within that context, and SI offers a conceptual perspective on how these capabilities may be enacted through critically informed human - AI collaboration. The double competence gap framework, in turn, directs attention to potential mismatches between these evolving competency requirements and the opportunities provided through initial dental education and continuing professional development.

From an educational perspective, this distinction has important implications. Dental education should not focus exclusively on teaching students how to operate specific AI tools. Instead, it should develop enduring professional competencies that enable students and practising dentists to evaluate AI-supported recommendations, recognise uncertainty and errors, justify clinical decisions independently, and determine when AI assistance is appropriate or inappropriate. Such competencies provide a foundation for responsible human - AI collaboration while preserving the central role of professional expertise and patient-centred care.

Previous publications have explored emerging applications of AI, professional judgement, and human - AI collaboration in healthcare and dental contexts (23–27). In addition, published competency frameworks for healthcare professionals highlight the importance of critically evaluating AI-based tools and understanding their implications for professional practice (28). Nevertheless, further empirical research is needed to establish how these competencies can best be developed and assessed in dental education.

### Assessing independent and AI-supported clinical competence

An important implication of the proposed double competence gap framework concerns how professional competence should be assessed in increasingly AI-enabled dental education. We propose distinguishing between two complementary dimensions of assessment: *independent clinical competence* and *AI-supported clinical competence*. This distinction is particularly relevant because responsible AI use presupposes sufficient professional knowledge and judgement to recognise when AI-generated information is incomplete, inaccurate, or clinically inappropriate.

*Independent clinical competence* refers to students' ability to demonstrate foundational clinical knowledge, diagnostic reasoning, ethical judgement, patient communication, and appropriate management decisions without relying on AI assistance. Such competence remains essential for patient safety, professional accountability, and situations in which AI tools are unavailable, unreliable, or inappropriate. Assessment should therefore continue to include supervised clinical examinations, scenario-based simulation, case-based reasoning, and other activities in which students independently identify relevant clinical information, consider differential diagnoses, and justify their decisions.

*AI-supported clinical competence,* by contrast, refers to students' ability to use AI systems critically, appropriately, and transparently as supplementary resources within professional practice. Assessment should examine whether students can identify suitable applications, evaluate AI-generated suggestions against clinical evidence and relevant guidelines, recognise hallucinations, uncertainty and potential bias, protect patient confidentiality, and justify whether AI-generated recommendations should be accepted, modified, or rejected. Importantly, the use of AI should not reduce students' responsibility for the final clinical judgement.

These two dimensions may be assessed through complementary activities using the same clinical scenario. For example, in a simulated dental trauma case, students could first independently assess the available clinical information, identify missing findings, consider differential diagnoses, and propose a management plan. In a subsequent AI-supported phase, students could critically examine an AI-generated recommendation, compare it with their initial reasoning and relevant clinical guidelines, and explain whether the AI output contributes to or potentially undermines the clinical decision. This approach would make both independent professional reasoning and critical human - AI interaction visible for assessment.

To ensure that assessment extends beyond agreement or disagreement with AI-generated outputs, students' clinical reasoning and critical appraisal should be evaluated against established clinical IADT-guidelines (International Association of Dental Traumatology) (21), relevant scientific evidence (22), and predefined assessment criteria developed by qualified dental educators and clinicians. In dental trauma scenarios, these criteria could address the identification of relevant clinical findings, diagnostic accuracy, recognition of uncertainty, appropriateness of proposed management, patient safety, and justification of clinical decisions. Structured assessment rubrics could be applied to both the independent and AI-supported phases, allowing educators to examine whether students identify clinically inappropriate AI recommendations, explain their reasoning, and make defensible decisions grounded in professional standards. The assessment should therefore prioritise the clinical quality and justification of students' decisions rather than their level of agreement with the AI system. The reliability and educational validity of such assessment procedures would require empirical evaluation.

The abovementioned distinction also highlights the importance of separating enduring professional competencies from tool-specific knowledge. Enduring competencies include clinical reasoning, evidence appraisal, ethical judgement, communication, patient safety, and professional accountability. Tool-specific knowledge concerns the functionality, limitations, and appropriate use of particular AI applications, which may change rapidly as technologies evolve. Consequently, assessment should prioritise transferable professional capabilities rather than proficiency with a particular AI model or interface (illustrated in Table 1).

**Table 1:** Assessment dimensions and revised assessment evidence in AI-enabled dental education.

Assessment dimension	Revised assessment evidence
Independent clinical competence	Unaided case-based assessment against predefined clinical criteria
AI literacy	Identification and justification of AI-generated errors, uncertainty, and bias against clinical evidence
AI-supported clinical competence	Clinical appraisal of AI recommendations using established guidelines and structured assessment rubrics
Reflective professional judgement	Evidence-based justification for accepting, modifying, or rejecting AI-generated recommendations

AI, artificial intelligence. The assessment dimensions and evidence are proposed and require empirical validation.

Within this framework, symbiotic intelligence provides a conceptual perspective on how human expertise and AI-supported information processing may complement one another without diminishing professional autonomy. However, whether such assessment approaches improve learning outcomes, clinical reasoning, or professional preparedness remains an empirical question requiring systematic educational evaluation.

Therefore, students' clinical reasoning and critical appraisal of AI-generated recommendations should be assessed against established clinical standards in every step of this Table 1, including the International Association of Dental Traumatology (IADT) (21) guidelines, rather than merely their agreement or disagreement with AI-generated outputs.

### From competency requirements to educational practice

Translating the proposed double competence gap framework into educational practice requires a systematic alignment between professional tasks, AI-related competencies, intended learning outcomes, educational activities, and assessment evidence. Rather than introducing AI as an isolated technological component, we propose embedding relevant AI-related competencies within existing clinical and educational contexts, while maintaining established standards of professional judgement, patient safety, and accountability.

For undergraduate dental students, educational activities could include supervised, case-based simulations in which students first demonstrate independent clinical reasoning and subsequently evaluate AI-generated recommendations against clinical findings and relevant guidelines. Postgraduate trainees may require more advanced, specialty-specific activities involving diagnostic uncertainty, complex cases, and critical evaluation of AI applications. For practising dentists, continuing professional development should address the appraisal of AI tools, clinical applicability, patient-data protection, and responsible implementation within local practice settings.

Dental educators represent an additional and essential target group. Their professional development should encompass the critical evaluation of AI systems, the design of appropriate learning activities and assessments, recognition of algorithmic errors and bias, and the ethical and pedagogical implications of AI-supported learning.

Across these groups, educational design should connect the professional task to the required competency, formulate observable learning outcomes, provide opportunities for supervised practice, and generate assessment evidence of what learners can demonstrate independently and with appropriate AI support. Such alignment may help institutions identify and address potential competency mismatches while avoiding an exclusive focus on proficiency with individual AI tools.

We propose this approach as a practical educational framework requiring contextual adaptation and empirical evaluation, rather than as a validated model of AI integration in dental education (see Table 2).

**Table 2:** Illustrative alignment of professional tasks, AI competencies, learning outcomes, educational activities, and assessment evidence in dental education and continuing professional development.

Target group and professional task	Required AI competency	Intended learning outcome	Educational activity	Assessment evidence
Undergraduate students: Dental trauma assessment	Critically evaluate AI-generated clinical recommendations	Identify missing information, recognise errors and justify a management decision	Two-stage case simulation, initially without AI and subsequently with AI support	Independent case assessment and documented critical appraisal of AI output
Postgraduate trainees: Complex specialty-specific diagnosis	Evaluate AI performance, limitations and uncertainty in specialised clinical contexts	Critically appraise AI-supported diagnostic suggestions and justify their clinical applicability	Specialty-specific case discussions and supervised simulations	Structured clinical reasoning and evaluation of AI-supported recommendations
Practising dentists: Evaluation of AI tools for clinical practice	Assess clinical validity, patient safety, data protection and professional accountability	Determine whether and under what conditions an AI tool is appropriate for local clinical use	Continuing professional development workshops and practice-based case evaluations	Documented appraisal of an AI application and justification of an implementation decision
Dental educators: Curriculum and assessment development	Evaluate AI tools and design pedagogically appropriate AI-supported activities	Design learning outcomes and assessments that distinguish independent from AI-supported competence	Faculty development workshops and collaborative assessment design	AI-related teaching plan and assessment rubric with explicit criteria

The educational recommendations presented in this article should be interpreted in relation to their respective levels of empirical support. Existing research provides evidence of emerging AI capabilities, variation in curricular integration, and the importance of clinically grounded professional competencies. However, the proposed double competence gap framework, the complementary assessment of independent and AI-supported clinical competence, and the use of domain-specific resources such as Si-DentAI represent conceptual or illustrative educational approaches whose effectiveness has not yet been established. Their potential contribution to clinical reasoning, AI literacy, and professional preparedness should therefore be examined through systematic piloting, appropriate comparison conditions, and empirical evaluation before broader implementation is recommended.

An illustrative application of this educational alignment (29) is dental trauma simulation, where students may first demonstrate independent clinical reasoning and subsequently critically examine AI-supported feedback. The Si-DentAI initiative described below provides an example of how such learning activities could be designed, although its educational effectiveness has not yet been established. Systematic piloting and evaluation would therefore be necessary before considering broader curricular implementation.

### Risks and limitations of AI integration in dental education

Although AI may offer opportunities to support learning and clinical decision-making, its integration into dental education also raises important pedagogical, ethical, and professional concerns. These risks should be considered alongside potential benefits rather than treated as secondary implementation challenges.

One concern is automation bias, whereby students or professionals may place excessive confidence in AI-generated recommendations, potentially reducing independent critical appraisal. Relatedly, inappropriate dependence on AI may contribute to deskilling, particularly if learners increasingly delegate diagnostic reasoning or clinical judgement to AI systems before developing sufficient foundational expertise. Generative AI may also produce plausible but inaccurate or fabricated clinical information, making independent verification against established clinical evidence and relevant guidelines essential.

Additional concerns include patient privacy and confidentiality, particularly when clinical information is entered into external AI systems, and inequitable algorithmic performance, where AI tools may perform differently across patient populations, clinical contexts, or underrepresented groups. Academic integrity also requires attention, as AI-supported assignments and assessments may obscure students' independent knowledge and reasoning unless expectations for appropriate AI use and disclosure are clearly established.

These challenges reinforce the importance of maintaining professional accountability and ensuring that AI-supported learning does not replace opportunities to develop independent clinical competence. They also provide a rationale for the complementary assessment approach proposed above, in which students demonstrate clinical reasoning both independently and when critically evaluating AI-supported information.

From this perspective, symbiotic intelligence should not be interpreted as an assumption that human–AI collaboration necessarily improves educational or clinical outcomes. Rather, it represents a conceptual approach in which potential benefits must be weighed against the risks of overreliance, error, bias, and diminished professional autonomy. Consequently, AI integration in dental education should proceed through carefully designed, supervised, and empirically evaluated educational activities.

### Faculty development and institutional readiness

Addressing the proposed double competence gap also requires attention to the competencies of dental educators. Effective AI integration cannot depend solely on students' technological familiarity or individual educators' initiatives. Faculty development should therefore be considered a cross-cutting component of both initial dental education and continuing professional development.

Dental educators need opportunities to develop competencies in critically evaluating AI systems, recognising hallucinations and algorithmic bias, designing pedagogically appropriate AI-supported learning activities, and assessing students' independent and AI-supported clinical reasoning. Such competencies also include understanding patient-data protection, academic integrity, professional accountability, and relevant institutional governance requirements.

We propose that faculty development should combine structured professional learning, collaborative curriculum design, and supervised experimentation with AI-supported educational activities. Institutions should establish clear guidance concerning approved AI applications, appropriate use of clinical data, transparency in assessment, and procedures for evaluating emerging technologies. Interdisciplinary collaboration among dental educators, clinicians, educational researchers, and specialists in AI and data protection may further support responsible implementation.

Importantly, faculty development should not be reduced to technical training in particular AI tools. It should strengthen educators' capacity to make informed pedagogical and professional decisions about when, how, and whether AI should be incorporated into dental education. Such institutional and professional preparedness may help address both dimensions of the proposed double competence gap, although its effectiveness requires systematic evaluation.

### Si-DentAI as an illustrative educational design

As an example of how AI literacy and symbiotic intelligence may be operationalised in dental education, we have developed Si-DentAI (Si-DentAI — Dental Trauma Education) (30), a domain-specific, AI-supported learning resource for case-based simulation in dental trauma education at the University of Bergen. The platform is designed to encourage students to engage in clinical reasoning through simulated patient interactions, guided questioning, and critical reflection on diagnostic and management decisions.

Rather than providing immediate answers, the intended pedagogical approach is to prompt students to identify relevant clinical information, consider differential diagnoses, justify their reasoning, and critically examine possible management strategies. An illustrative case involving avulsion of an immature permanent incisor demonstrates how AI-supported questioning may be incorporated into a simulated dental trauma scenario (Supplementary Material 1).

Within the proposed double competence gap framework, Si-DentAI illustrates a possible educational approach to developing profession-specific AI literacy while preserving the importance of independent clinical reasoning and professional judgement. However, the platform has not yet been systematically evaluated for its effects on student learning, diagnostic reasoning, clinical performance, or patient-related outcomes. Its inclusion in this article should therefore be understood as an example of educational design rather than evidence of educational effectiveness.

Before considering broader curricular implementation, including potential integration into compulsory educational activities, such resources should undergo systematic piloting and evaluation. Relevant outcomes may include students' clinical reasoning, ability to identify AI-generated errors, critical appraisal of AI-supported recommendations, perceived usefulness, and the extent to which AI-supported learning complements rather than replaces independent professional competence. Evaluation should also address patient-data protection, algorithmic bias, educator oversight, and the reliability of AI-generated feedback.

Accordingly, we propose that domain-specific AI-supported learning resources such as Si-DentAI may be explored through carefully supervised educational pilots, with decisions concerning wider implementation guided by empirical evidence, educational relevance, and institutional governance requirements.

AI-supported technologies may also have implications for access to health information and professional services, particularly in settings where geographical distance, limited service availability, or other structural barriers affect access to care. However, the extent to which patients use generative AI for self-assessment or health-related decision-making in such contexts, and whether this improves or undermines health outcomes, remains uncertain.

In dentistry, these developments raise questions about how professionals should respond when patients present AI-generated information or recommendations during clinical consultations. Dental education may therefore need to prepare students to critically evaluate such information, communicate its limitations, and support informed, patient-centred decision-making. These potential implications should be investigated empirically rather than assumed to represent an established transformation of healthcare delivery (20).

Within this conceptual perspective, symbiotic intelligence provides a rationale for exploring AI-supported educational activities in which students retain responsibility for clinical reasoning while using AI as a critically evaluated learning resource.

### Disciplinary scope and generalisability

Although the proposed double competence gap framework may have relevance across several healthcare professions, the present article focuses primarily on dental education. Dentistry provides a relevant context because AI-supported technologies are increasingly being explored in diagnostic assessment, clinical decision support, patient communication, and professional learning. The Si-DentAI initiative further illustrates how AI-related competencies may be addressed through domain-specific educational design.

Nevertheless, the professional tasks, regulatory requirements, clinical responsibilities, and educational structures of dentistry differ from those of medicine, psychology, and other healthcare professions. Consequently, the proposed framework should not be assumed to apply uniformly across professional domains or national contexts.

We therefore present the double competence gap as a potentially transferable conceptual framework whose applicability, dimensions, and educational implications require further empirical investigation in different professional education settings.

Figure 1 summarises the proposed double competence gap framework and its educational implications. The model distinguishes between potential mismatches arising during initial professional education and those emerging as practising dentists encounter evolving AI-related competency requirements. AI literacy, situated within the broader concept of Professional Digital Competence, represents an important competency dimension, while symbiotic intelligence provides a conceptual perspective on responsible human–AI collaboration. The framework also identifies curriculum alignment, assessment, faculty development, and continuing professional development as possible educational responses. Importantly, the model is intended to guide conceptual clarification and future empirical investigation rather than to demonstrate the existence, magnitude, or causal mechanisms of the proposed competence gaps.

**Figure 1:**
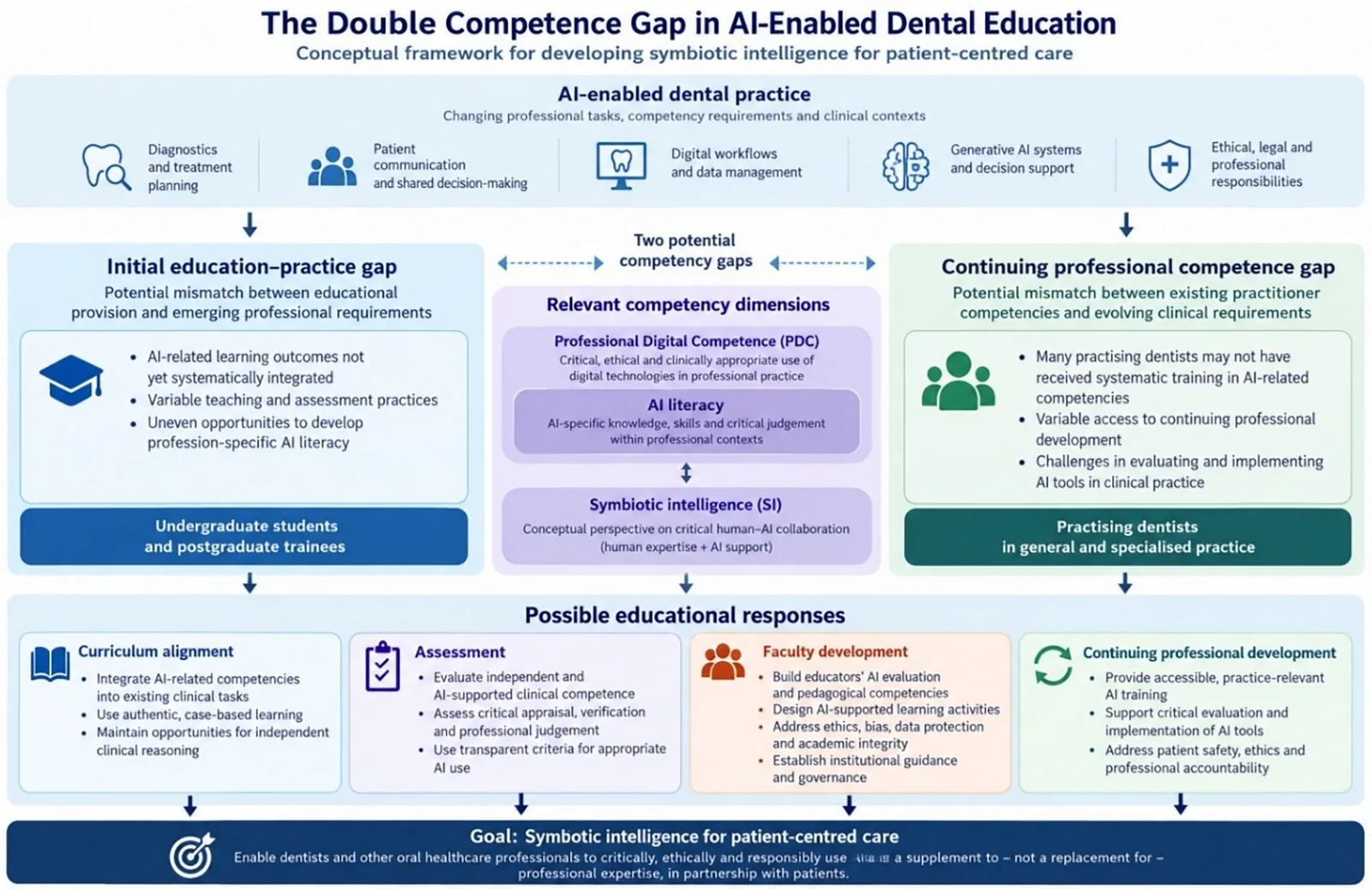
The proposed double competence gap framework in AI-enabled dental education.

The framework distinguishes between two potential competency mismatches: (1) the initial education–practice gap, concerning the alignment between dental education and emerging professional competency requirements; and (2) the continuing professional competence gap, concerning the alignment between practising dentists' existing competencies and evolving clinical requirements. Professional Digital Competence (PDC) and AI literacy represent relevant competency dimensions, while symbiotic intelligence provides a conceptual perspective on critical human–AI collaboration. Curriculum alignment, assessment, faculty development, and continuing professional development are proposed as potential educational responses. The framework is conceptual and requires empirical investigation; the relationships illustrated should not be interpreted as established causal effects.

The proposed framework may provide a basis for reconsidering educational priorities, professional competencies, and responsibilities in light of evolving scientific knowledge (3), contextual experiences from dental practice (31–33), and emerging AI capabilities. Such reconsideration should aim to ensure that critical, evidence-informed, and ethically responsible AI use becomes an integral part of the professional knowledge infrastructure. The increasing relevance of AI in dental practice also raises questions about whether existing professional regulations, institutional guidelines, and continuing professional development arrangements adequately address emerging requirements concerning AI-related competencies, patient safety, data protection, and professional accountability. Rather than presuming that legislative amendments are necessary, we suggest that the adequacy of existing regulatory and professional frameworks should be systematically evaluated as AI-supported technologies become more widely integrated into dental practice (34).

Within this context, addressing the potential double competence gap calls for a coherent educational approach in which Professional Digital Competence provides a broad competency foundation, AI literacy addresses the specific requirements of critically understanding and evaluating AI technologies, and symbiotic intelligence offers a conceptual perspective on responsible human–AI collaboration. Together, these perspectives may inform the development of educational strategies that preserve independent clinical judgement while supporting the critical and appropriate use of AI in patient-centred dental care.

## Conclusion

The increasing integration of AI into dental practice raises important questions about how professional education can maintain alignment with evolving clinical and technological requirements. In this Opinion article, we have proposed the double competence gap as a conceptual framework for examining potential mismatches in both initial dental education and the continuing professional development of practising dentists.

Addressing these challenges requires more than familiarity with AI tools. Dental education should preserve foundational clinical knowledge, independent diagnostic reasoning, ethical judgement, and professional accountability while developing the competencies needed to critically evaluate and appropriately use AI-supported information. From this perspective, symbiotic intelligence offers a conceptual approach to human–AI collaboration in which AI may augment, but should not replace, professional expertise and patient-centred clinical judgement.

We propose that curriculum alignment, complementary assessment of independent and AI-supported competence, faculty development, and continuing professional development represent relevant educational responses. Domain-specific resources such as Si-DentAI may provide opportunities to explore these approaches through supervised scenario-based simulation, but their educational effectiveness must be established before broader implementation is recommended.

The existence and extent of the proposed competence gaps, and the effectiveness of educational strategies intended to address them, remain empirical questions. Future research should therefore investigate how AI-related competencies are developed, assessed, and maintained across different educational and professional contexts. The overarching goal is not simply to increase AI adoption, but to prepare dental professionals to engage with emerging technologies critically, ethically, and responsibly in support of patient-centred care.
