# Supplementary material for: AI and the double competence gap in dental education: towards symbiotic intelligence
Source: Front Dent Med. 2026 Oct 29;7:1965756. doi: 10.3389/fdmed.2026.1965756 (PMC13755260; doi:10.3389/fdmed.2026.1965756)
Supplement: Supplementary file 1 [file Supplementaryfile1.pdf]

Supplementary Material 1

AI and the double competence gap in dental education: towards symbiotic intelligence

Krumsvik RJ, Klock K, Bratteberg M. Frontiers in Dental Medicine (2026). doi: 10.3389/fdmed.2026.1965756

Supplementary case example: Avulsion of an immature permanent incisor

This supplementary material illustrates how Si-DentAI is intended to support case-based learning in dental trauma education. The simulated case concerns an eight-year-old child whose upper right central incisor (tooth 11) was knocked out in a fall at a school playground approximately 45 minutes before assessment. The tooth was found within a few minutes, rinsed briefly and placed in milk by the school nurse. Students are asked to take the history from a simulated patient (the written record is not shown until the student has done so), review the examination findings, and reason through the significance of the open apex and the short extra-alveolar dry time before proposing management.

The screenshots (Supplementary Figures S1–S3) show the clinical workspace, a history-taking exchange with the simulated patient, and a two-turn exchange with the AI tutor. They illustrate the platform's educational design; they do not establish its effectiveness or present a complete clinical assessment. All screenshots were captured from a logged-in student session in the production environment on 22 October 2026 (text mode, English). The patient and tutor responses are generated by a general-purpose language model (OpenAI GPT-6, accessed via API) constrained by role-specific system prompts, the structured case record and retrieval from the IADT 2020 guidelines; they are reproduced verbatim and have not been edited. A public description of Si-DentAI is available at https://si-dent-ai.vercel.app/about. The cases themselves are used in a login-protected teaching environment (https://si-dent-ai.vercel.app/login) and are not openly accessible; the figures below show the case as a student sees it.

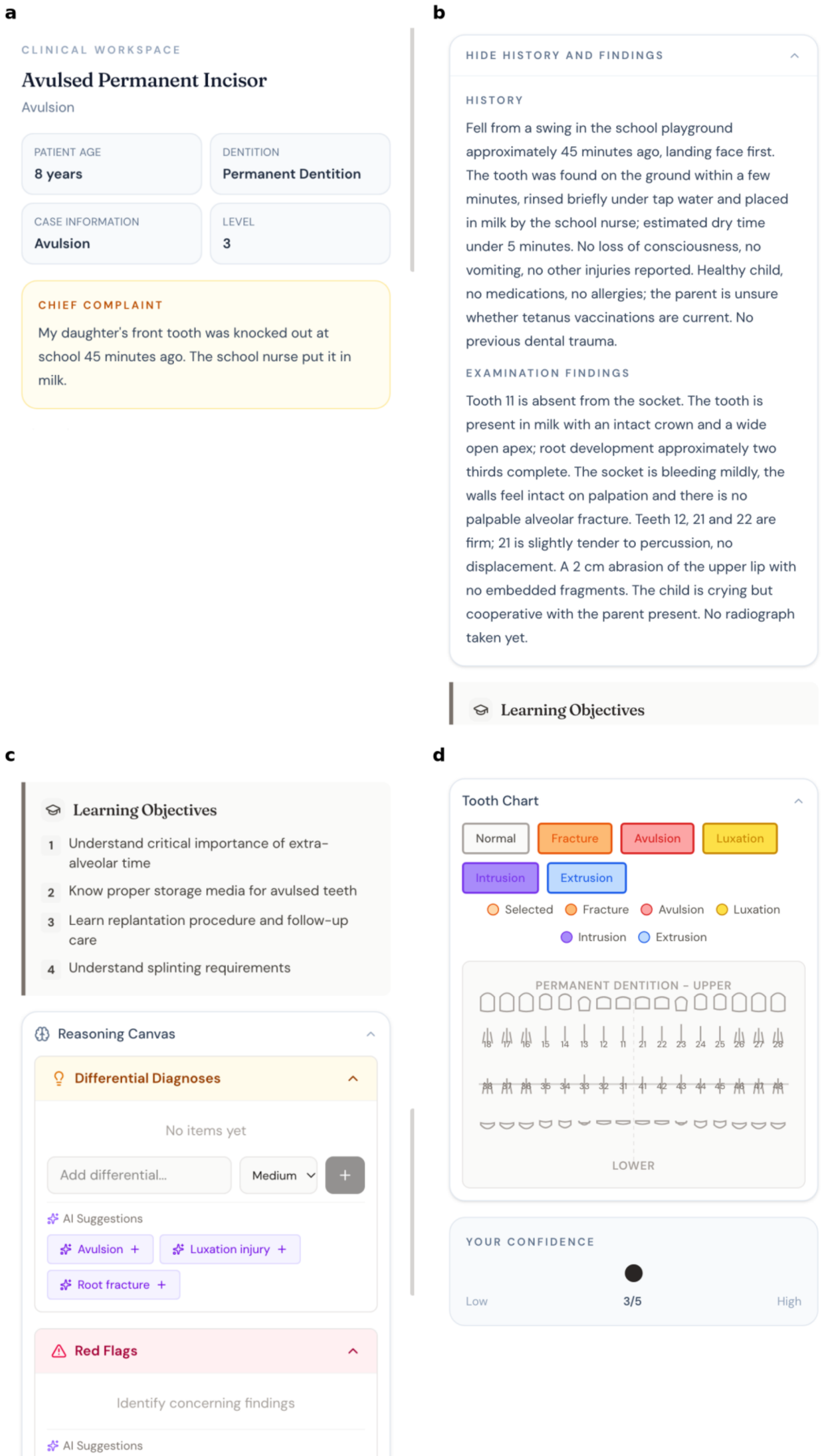

a
CLINICAL WORKSPACE
Avulsed Permanent Incisor
Avulsion
PATIENT AGE
8 years
DENTITION
Permanent Dentition
CASE INFORMATION
Avulsion
LEVEL
3
CHIEF COMPLAINT
My daughter's front tooth was knocked out at school 45 minutes ago. The school nurse put it in milk.
b
HIDE HISTORY AND FINDINGS
HISTORY
Fell from a swing in the school playground approximately 45 minutes ago, landing face first. The tooth was found on the ground within a few minutes, rinsed briefly under tap water and placed in milk by the school nurse; estimated dry time under 5 minutes. No loss of consciousness, no vomiting, no other injuries reported. Healthy child, no medications, no allergies; the parent is unsure whether tetanus vaccinations are current. No previous dental trauma.
EXAMINATION FINDINGS
Tooth 11 is absent from the socket. The tooth is present in milk with an intact crown and a wide open apex; root development approximately two thirds complete. The socket is bleeding mildly, the walls feel intact on palpation and there is no palpable alveolar fracture. Teeth 12, 21 and 22 are firm; 21 is slightly tender to percussion, no displacement. A 2 cm abrasion of the upper lip with no embedded fragments. The child is crying but cooperative with the parent present. No radiograph taken yet.
Learning Objectives
c
Learning Objectives
1 Understand critical importance of extra-alveolar time
2 Know proper storage media for avulsed teeth
3 Learn replantation procedure and follow-up care
4 Understand splinting requirements
Reasoning Canvas
Differential Diagnoses
No items yet
Add differential...
Medium
+
AI Suggestions
Avulsion +
Luxation injury +
Root fracture +
Red Flags
Identify concerning findings
AI Suggestions
d
Tooth Chart
Normal
Fracture
Avulsion
Luxation
Intrusion
Extrusion
Selected
Fracture
Avulsion
Luxation
Intrusion
Extrusion
PERMANENT DENTITION – UPPER
LOWER
YOUR CONFIDENCE
Low
3/5
High

Supplementary Figure S1. The clinical workspace in Si-DentAI. (a) Case header with patient age, dentition, injury category and difficulty level, and the chief complaint in the parent's own words. In the application a clinical photograph is shown below the chief complaint; it is omitted here. (b) The written history and examination findings, which are hidden until the student has interviewed the simulated patient. (c) Learning objectives and the Reasoning Canvas, where the student records differential diagnoses, red flags and next steps; AI-generated suggestions can be added or ignored. (d) The tooth chart, on which the student marks the injured tooth and injury type, and a self-rated confidence scale. The expected diagnosis is stored with the case but is not visible to the student.

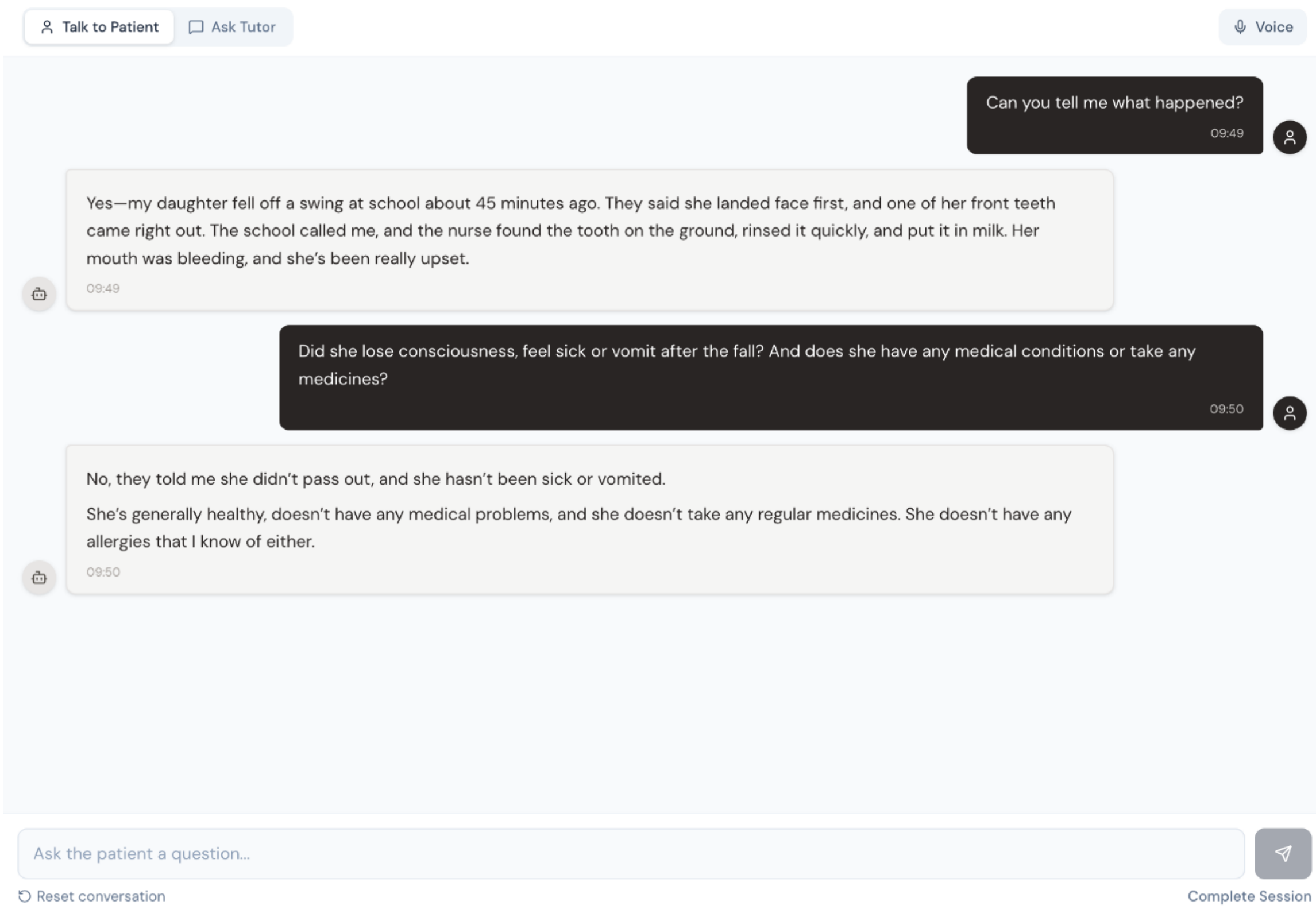

Supplementary Figure S2. Simulated patient interview in Si-DentAI. In the Talk to Patient workspace the student practises history-taking. The simulated patient (here the parent of the child) answers only what is asked, in character, and does not volunteer clinical findings, diagnoses or treatment suggestions. The first question was selected from the suggested prompts; the second was typed by the student. Separate Ask Tutor and voice options are visible in the interface.

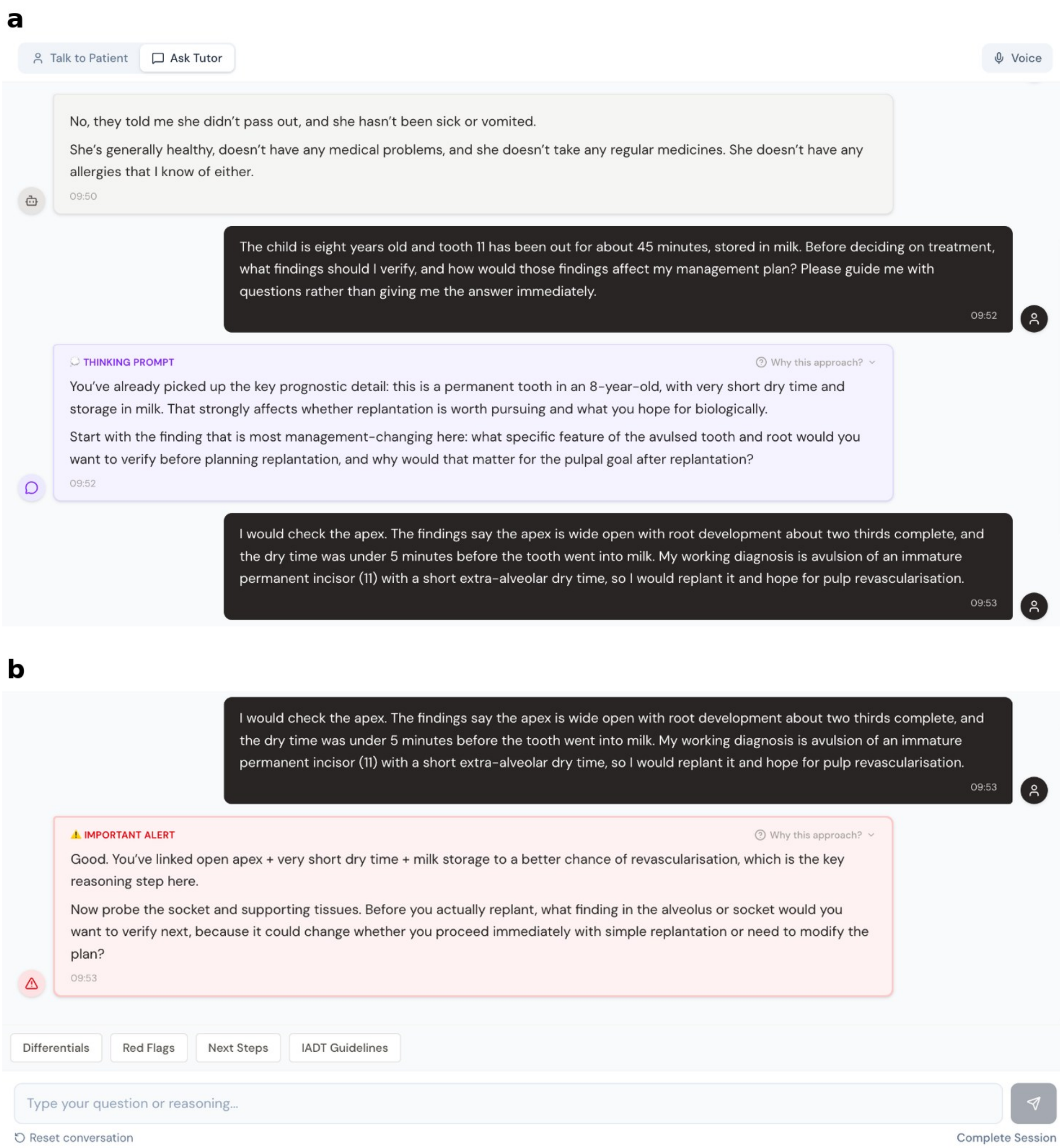

Supplementary Figure S3. Example of a student–tutor exchange in Si-DentAI. (a) The student asks which findings should be verified before deciding on treatment and asks to be guided by questions rather than given the answer. The tutor acknowledges the prognostic details the student has already identified (permanent tooth, short dry time, storage in milk) and asks a single question directing attention to the feature of the avulsed tooth that most affects the pulpal goal of replantation. (b) The student commits to a working diagnosis and a management intention; the tutor confirms the reasoning step and asks one further question about the socket and supporting tissues before replantation. Across the exchange the tutor does not state a management plan; each turn ends with exactly one question that moves the reasoning one step forward. The coloured labels above the tutor messages ("Thinking prompt", "Important alert") are applied by the interface on the basis of the message content; "Why this

approach?" opens a short explanation of the pedagogical intent of the turn. Shortcuts to Differentials, Red Flags, Next Steps and IADT Guidelines provide additional routes into the case.

Verbatim transcript of the exchange shown in Supplementary Figure S3

Student: The child is eight years old and tooth 11 has been out for about 45 minutes, stored in milk. Before deciding on treatment, what findings should I verify, and how would those findings affect my management plan? Please guide me with questions rather than giving me the answer immediately.

Tutor: You've already picked up the key prognostic detail: this is a permanent tooth in an 8-year-old, with very short dry time and storage in milk. That strongly affects whether replantation is worth pursuing and what you hope for biologically. Start with the finding that is most management-changing here: what specific feature of the avulsed tooth and root would you want to verify before planning replantation, and why would that matter for the pulpal goal after replantation?

Student: I would check the apex. The findings say the apex is wide open with root development about two thirds complete, and the dry time was under 5 minutes before the tooth went into milk. My working diagnosis is avulsion of an immature permanent incisor (11) with a short extra-alveolar dry time, so I would replant it and hope for pulp revascularisation.

Tutor: Good. You've linked open apex + very short dry time + milk storage to a better chance of revascularisation, which is the key reasoning step here. Now probe the socket and supporting tissues. Before you actually replant, what finding in the alveolus or socket would you want to verify next, because it could change whether you proceed immediately with simple replantation or need to modify the plan?
